# Readability and Quality of Chatbot Responses to Periodontal Patient Queries: A Cross‐Sectional Evaluation of Three Publicly Accessible Large Language Models

**DOI:** 10.1155/ijod/3188084

**Published:** 2026-06-27

**Authors:** Nicola Alberto Valente, Lorenzo Floris, Chiara Cinquini, Zuhair S. Natto, Lorenzo Mordini

**Affiliations:** ^1^ Division of Periodontology, School of Dental Medicine, Department of Surgical Sciences, Faculty of Medicine and Surgery, University of Cagliari, Cagliari, Italy, unica.it; ^2^ College of Dentistry, American University of Iraq, Baghdad, Iraq, auib.edu.iq; ^3^ Department of Surgical Medical Molecular and Critical Area Pathology, University of Pisa, Pisa, Italy, unipi.it; ^4^ Department of Dental Public Health, Faculty of Dentistry, King Abdulaziz University, Jeddah, Saudi Arabia, kau.edu.sa; ^5^ Department of Periodontology, School of Dental Medicine, Tufts University, Boston, Massachusetts, USA, tufts.edu

## Abstract

**Objective:**

The increasing use of large language models (LLMs) as sources of health information raises concerns regarding the quality and readability of patient‐directed content. This study aimed to evaluate and compare the readability and quality of responses generated by three publicly accessible LLMs, ChatGPT‐4o, Google Gemini 2.0 Flash, and DeepSeek‐R1, to frequently asked patient questions related to periodontology.

**Materials and Methods:**

In this cross‐sectional study, 48 real‐world periodontal questions were retrieved from Reddit and Quora and entered verbatim into each chatbot (February 2025, default settings). Readability was assessed using Flesch Reading Ease (FRE) and Flesch–Kincaid Grade Level (FKGL). Quality and accuracy were evaluated using the Quality Analysis of Medical Artificial Intelligence (QAMAI) tool by three independent expert periodontists. Mean scores were compared using one‐way ANOVA with Tukey post‐hoc tests (*α* = 0.05).

**Results:**

FKGL differed significantly among models (*p* = 0.007), with ChatGPT‐4o producing the most complex text (11.18 ± 1.55) compared to DeepSeek‐R1 (9.84 ± 1.78) and Gemini (9.91 ± 1.89). FRE did not differ significantly (*p* = 0.144), although all scores (42.37–49.55) fell within the “difficult” readability range. Mean QAMAI scores were comparable across models (*p* = 0.078), ranging from 18.31 ± 1.90 (Gemini) to 19.10 ± 1.78 (DeepSeek‐R1), placing the mean scores at the lower end of the predefined “good quality” range. Subgroup analysis revealed significant readability differences only within the “pockets” domain (*p* = 0.001), while quality remained broadly consistent across topics.

**Conclusions:**

All evaluated LLMs produced responses with mean QAMAI scores at the lower end of the predefined good‐quality range; however, their readability exceeded recommended standards for public health materials. While LLMs may serve as supplementary educational tools, language simplification strategies and continued professional oversight remain essential to ensure safe and accessible patient information.

## 1. Introduction

In recent years, the integration of artificial intelligence (AI) and large language models (LLMs) into healthcare has marked a paradigm shift, fundamentally transforming diagnostic protocols, treatment planning, and administrative workflows [1, 2]. In dentistry, AI applications have expanded rapidly, ranging from radiographic analysis for caries detection and bone loss assessment to complex decision‐making in prosthodontics and oral surgery [1, 3–5]. Among these technologies, generative AI models, such as ChatGPT (OpenAI), Google Gemini (formerly Bard), and newer iterations like DeepSeek, have demonstrated remarkable capabilities in processing natural language and generating human‐like responses, making them accessible tools not only for clinicians but also for patients seeking medical information [6, 7].

Traditionally, patients relied on search engines or online forums to find answers regarding their symptoms and treatment options [6, 8]. However, the landscape of information‐seeking behavior is evolving; patients are increasingly turning to AI chatbots for immediate, synthesized answers to complex health queries, bypassing the need to navigate multiple websites [6, 8, 9]. This trend is particularly relevant in dentistry, where anxiety and cognitive burden can hinder a patient’s ability to process information provided during clinical consultations [9, 10]. Consequently, AI chatbots are becoming “virtual assistants” for patient education, offering explanations on conditions, postoperative care, and therapeutic alternatives [11–13].

Despite their potential, the widespread adoption of LLMs raises significant concerns regarding the accuracy, reliability, and readability of the generated content [1, 14, 15]. LLMs are known to suffer from “hallucinations,” generating plausible but factually incorrect information, which poses a substantial risk to patient safety [1, 13, 16]. Furthermore, for health information to be effective, it must adhere to health literacy standards; complex medical jargon can alienate patients, leading to poor treatment compliance and uninformed decision‐making [8, 10]. Previous studies evaluating AI responses in various dental specialties—such as oral surgery, dental trauma, and implantology—have highlighted variability in performance, often noting that while accuracy may be acceptable, the readability levels frequently exceed the recommended standards for general public comprehension [6, 10, 12].

In the field of Periodontology, effective patient communication is critical. Periodontitis is a chronic condition where long‐term success relies heavily on patient compliance, understanding of risk factors, and adherence to maintenance protocols [17–19]. Misinformation regarding gum recession, pocket reduction, or regenerative procedures can lead to unrealistic expectations or the neglect of necessary treatment [9, 16]. While recent studies have assessed the performance of AI in answering board‐style examinations or student queries in periodontology, there is a paucity of research evaluating how the latest generation of LLMs responds to real‐world patient inquiries [16, 20, 21].

Therefore, the primary aim of this study was to evaluate and compare the readability of responses generated by three advanced LLMs—ChatGPT‐4o, Google Gemini 2.0 Flash, and DeepSeek‐R1—to frequently asked patient questions related to periodontology. The secondary aim was to assess and compare the quality and accuracy of these responses using the QAMAI metric.

## 2. Materials and Methods

This study was designed as a cross‐sectional analysis and reported in accordance with the STROBE recommendations for observational studies. Given the involvement of LLMs, TRIPOD‐LLM recommendations were also considered where applicable [22, 23]. Institutional Review Board (IRB) approval was not required because the study used only publicly available online questions, did not involve interaction with human participants, did not collect private or identifiable information, and reported no usernames, profile data, or direct identifiers. Although the questions were retrieved from public forums where user profiles may be visible, all data were anonymized, and no personal identity information regarding the users who posted the queries is reported in this article.

### 2.1. Data Source, Question Selection, and LLM Processing

A total of 48 frequently asked patient questions related to periodontology were sourced from the online question‐and‐answer platforms (Reddit and Quora) to reflect real‐world information‐seeking behaviors. Searches were conducted in February 2025 using the standard search interfaces available on each platform at the time. On Reddit, searches were restricted to the r/askdentists community and used the predefined keywords “periodontitis,” “pockets,” “periodontal regeneration,” and “gum recession.” On Quora, the search term “periodontology” was used. No additional manual filters based on date, popularity, or user engagement were applied.

Candidate questions were independently screened by two investigators according to predefined eligibility criteria. Inclusion criteria were: (1) relevance to periodontal diagnosis, prognosis, treatment, or maintenance; (2) patient‐oriented wording; (3) sufficient clarity to allow interpretation without images; and (4) non‐duplication of a previously selected questions. Exclusion criteria were as follows: (1) questions requiring interpretation of radiographs or clinical photographs; (2) posts dominated by unrelated medical or dental conditions; (3) duplicate or near‐duplicate questions; (4) questions containing identifiable personal information that could not be removed; and (5) questions that were too vague to permit a meaningful answer. Disagreements were resolved by discussion with a senior periodontist.

Consecutive eligible questions were retained within each keyword domain. The final number of 48 questions was determined pragmatically to provide coverage of the main periodontal domains while maintaining the feasibility of independent assessment by three expert periodontists. The final dataset comprised Reddit questions identified using the keywords “periodontitis” (*n* = 11), “pockets” (*n* = 11), “periodontal regeneration” (*n* = 4), and “gum recession” (*n* = 9), together with 13 Quora questions identified using the term “periodontology.”

**Figure 1:**
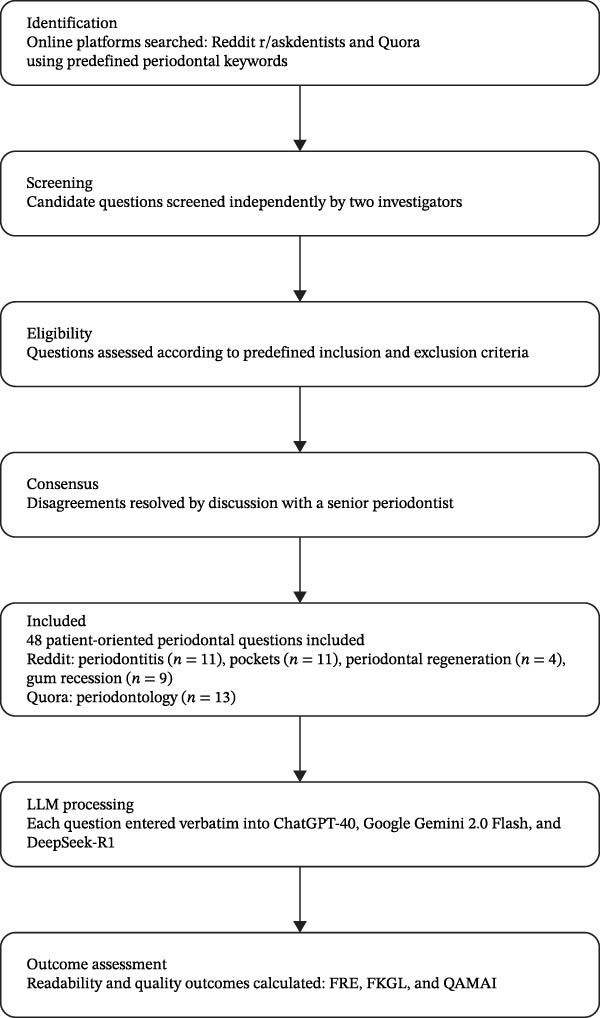
Flow diagram of the pragmatic question‐selection and LLM‐processing workflow. Abbreviations: FKGL, Flesch–Kincaid Grade Level; FRE, Flesch Reading Ease; QAMAI, Quality Analysis of Medical Artificial Intelligence; LLM, large language models.

Because the study was designed to construct a pragmatic dataset of real‐world patient questions rather than to perform a systematic content analysis of Reddit and Quora, the total numbers of posts displayed, screened, excluded, and removed as duplicates were not prospectively recorded. A question‐selection flow diagram is provided as Figure 1.

Each question was entered verbatim into ChatGPT‐4o (OpenAI), Google Gemini 2.0 Flash, and DeepSeek‐R1, without rephrasing, correction, or normalization of the original patient wording. Each question was submitted in a separate new chat session, with no preceding conversational context, no additional prompting, and no follow‐up questions. All responses were generated using the publicly accessible web interfaces of the three LLMs under default settings during the same data‐collection period in February 2025 and were recorded immediately after the initial response. The list of questions and responses from each LLM is included in Table S1.

### 2.2. Response Evaluation

The generated responses were evaluated for readability using established formulas and for quality using a validated tool specifically designed for medical AI output.

Readability was assessed using the Flesch Reading Ease (FRE) and the Flesch‐Kincaid Grade Level (FKGL) [24, 25]. These tests evaluate text difficulty by analyzing two core factors: sentence length (average number of words per sentence) and word length (average number of syllables per word), based on the premise that longer sentences and polysyllabic words increase complexity.

1. FRE: This metric rates text on a scale from 0 to 100. In contrast to most readability scores, a higher FRE score indicates material that is easier to read.
  • Scoring Interpretation: A score between 60 and 70 is considered “plain English” (standard for web content) easily understood by 13‐ to 15‐year‐old students (8th and 9th grade). Conversely, scores between 0 and 30 are classified as “very difficult” (academic or complex technical material) and are best understood by university graduates.
2. FKGL: Developed by the US Navy to simplify the interpretation of readability scores for technical manuals, this formula translates the readability score into a U.S. school grade level ranging from 0 to 18.
  • Scoring Interpretation: The score corresponds to the years of education required to understand the text. For example, an FKGL of 8.0 indicates that the text is understandable by an average 8th‐grade student (ages 13–14). For health information intended for the general public, a grade level of around 8 is recommended to ensure engagement and comprehension.

QAMAI was selected because it was specifically developed and validated for the assessment of health information generated by AI platforms. Unlike DISCERN, from which QAMAI has been developed and which was originally designed for written consumer health information and includes elements that do not fully translate to AI‐generated responses, QAMAI evaluates accuracy, clarity, relevance, completeness, sources, and usefulness as a single quality construct.

The quality of the AI‐generated responses was evaluated using the Quality Analysis of Medical Artificial Intelligence (QAMAI) tool [26]. Developed and validated by Vaira et al. [26] specifically to address the limitations of previous tools like DISCERN [27] when applied to AI, QAMAI assesses health information across a unidimensional construct of quality defined by six specific items:

1. Accuracy: The information provided is accurate and up‐to‐date.
2. Clarity: The answer is clear and comprehensible in terms of language and scientific terminology.
3. Relevance: The information is relevant and directly answers the question posed.
4. Completeness: The response adequately covers all aspects of the question, including areas of uncertainty.
5. Sources: The response provides reliable sources and references to support the health information presented.
6. Usefulness: The response met the user’s health information needs.

Each item was rated by the evaluators on a 5‐point Likert scale (1 = Strongly Disagree, 2 = Disagree, 3 = Neutral, 4 = Agree, and 5 = Strongly Agree). The individual item scores were summed to produce a total QAMAI score ranging from 6 to 30, classified as follows:

• 6–11 (poor quality): Largely unreliable or incomplete information requiring immediate improvement.
• 12–17 (fair quality): Contains some useful information but significant areas for improvement.
• 18–23 (good quality): Mostly reliable and complete, with some areas for refinement.
• 24–29 (very good quality): Reliable and complete in most areas.
• 30 (excellent quality): Highly reliable and complete information.

Three independent expert periodontists assessed the responses. To ensure a diverse and rigorous standard of evaluation, the panel consisted of two American Board of Periodontology certified and trained periodontists and one European‐trained periodontist. Prior to the evaluation process, the three evaluators reviewed the European Federation of Periodontology (EFP) S3 Level Clinical Practice Guidelines for the treatment of periodontitis and periodontal conditions to ensure a standardized evaluation framework based on the most recent evidence [28, 29]. For every response to the same question, the mean of the scores assigned by the three evaluators was calculated to determine the final value. Inter‐rater reliability for the QAMAI total score was assessed using a two‐way random‐effects intraclass correlation coefficient (ICC) for absolute agreement.

### 2.3. Statistical Analysis

Descriptive statistics (mean and standard deviation) were computed for each metric by model and keyword group. Differences in mean FKGL, FRE, and QAMAI across the three models were evaluated using one‐way analysis of variance (ANOVA), with model type as the independent variable and each readability metric as the dependent variable. Prior to conducting ANOVA, assumptions of normality and homogeneity of variances were assessed using diagnostic plots and Levene’s test. For significant ANOVA results, Tukey’s Honest Significant Difference (HSD) post‐hoc tests were conducted to identify pairwise differences between models. Subgroup analyses by keyword domain were considered exploratory. No formal adjustment for multiple comparisons was applied; therefore, subgroup‐specific *p* values were interpreted cautiously and were considered primarily descriptive and hypothesis‐generating. Inter‐rater reliability for QAMAI scoring was assessed using the ICC because the same set of responses was independently rated by three evaluators. All statistical tests were two‐tailed, with an alpha level of 0.05 denoting statistical significance. Analyses were performed using SPSS statistical software (version 23, IBM Corp., Armonk, NY, USA).

## 3. Results

### 3.1. Readability (FKGL and FRE)

The overall readability outcomes are summarized in Table 1. FKGL differed significantly across models (*p* = 0.007), with ChatGPT‐4o generating the most complex text (11.18 ± 1.55), while DeepSeek‐R1 (9.84 ± 1.78) and Gemini (9.91 ± 1.89) produced lower grade‐level outputs. In contrast, FRE scores did not significantly differ among LLMs (*p* = 0.144), although ChatGPT‐4o showed numerically higher FRE values (49.55 ± 9.78) than DeepSeek‐R1 (44.67 ± 11.22) and Gemini (42.37 ± 11.42).

**Table 1:** Comparison of FRE, FKGL, and QAMAI scores according to AI chatbots.

Metric	ChatGPT‐4o	DeepSeek‐R1	Google GEMINI	*p*‐Value
FKGL score	11.18 ± 1.55	9.84 ± 1.78	9.91 ± 1.89	0.007 ^∗^
FRE score	49.55 ± 9.78	44.67 ± 11.22	42.37 ± 11.42	0.144
QAMAI score	18.49 ± 2.01	19.10 ± 1.78	18.31 ± 1.90	0.078

^∗^ *p* value <0.05.

In absolute terms, the FKGL values clustered around grades 10–11 (min. 9.84, max. 11.18), indicating that, on average, the generated content required a high‐school reading level.

The direction of the between‐model difference was consistent: ChatGPT‐4o tended to use longer sentences and/or more complex vocabulary, pushing the average FKGL above 11, whereas DeepSeek‐R1 and Gemini were closer to 10.

For FRE, the overall means, ranging between 42.37 and 49.55, fall largely within the “difficult” readability band (i.e., not “plain English”). However, wide standard deviations (≈10–11 points) were registered.

### 3.2. Quality of AI‐Generated Information (QAMAI)

Mean QAMAI scores were broadly comparable across LLMs (*p* = 0.078). DeepSeek‐R1 showed the highest numerical QAMAI score (19.10 ± 1.78), followed by ChatGPT‐4o (18.49 ± 2.01) and Gemini (18.31 ± 1.90), indicating overall similar quality levels across platforms. Inter‐rater reliability for QAMAI total scores showed good agreement among evaluators, with an ICC of 0.82 (95% CI: 0.74–0.88; *p* < 0.001).

Using the predefined QAMAI score bands (18–23 = “Ggoodquality”), the overall mean scores for all three models fall within the “Ggood category. However, averages were concentrated around 18–19, close to the lower boundary of this category and should therefore be interpreted as borderline good quality rather than as evidence of consistently high‐quality patient information. The magnitude of the SDs (≈1.8–2.0) indicates moderate dispersion in perceived quality across questions.

### 3.3. Subgroup Analysis by Keyword Domain

Exploratory subgroup analyses by keyword category are reported in Table 2. A nominally significant between‐model difference was observed only for FKGL within the Reddit “pockets” subgroup (*p* = 0.001), showing higher complexity for ChatGPT‐4o (11.18 ± 1.08) than for DeepSeek‐R1 (9.57 ± 1.28) and Gemini (9.21 ± 0.83). Given the small sample sizes of some subgroups and the absence of adjustment for multiple comparisons, these findings should be interpreted cautiously. For the remaining keyword domains (“periodontitis,” “periodontal regeneration,” “gum recession,” and Quora “periodontology”), no statistically significant differences were observed across models for FKGL, FRE, or QAMAI (all *p* > 0.05), despite some numerical variability.

**Table 2:** Comparison of FRE, FKGL, and QAMAI scores based on the keywords.

Metric	ChatGPT‐4o	DeepSeek‐R1	Google GEMINI	*p*‐Value
Reddit—“pockets”				
FKGL score	11.18 ± 1.08	9.57 ± 1.28	9.21 ± 0.83	0.001 ^∗^
FRE score	49.86 ± 5.13	47.54 ± 8.30	48.12 ± 6.14	0.624
QAMAI (mean ± SD)	17.59 ± 2.54	17.14 ± 2.21	18.09 ± 2.63	0.724
Reddit—“periodontitis”				
FKGL score	10.61 ± 1.60	9.21 ± 1.58	9.25 ± 0.84	0.285
FRE score	51.77 ± 7.51	51.75 ± 8.71	43.47 ± 8.16	0.080
QAMAI score	20.20 ± 1.33	19.41 ± 1.63	20.11 ± 1.47	0.879
Reddit—“periodontal regeneration”				
FKGL score	11.73 ± 0.70	11.40 ± 0.25	11.10 ± 0.30	0.404
FRE score	44.30 ± 5.57	39.60 ± 3.81	37.30 ± 1.67	0.082
QAMAI score	19.75 ± 1.26	19.00 ± 0.82	20.38 ± 1.29	0.194
Reddit—“gum recession”				
FKGL score	9.91 ± 1.66	9.25 ± 1.35	8.22 ± 1.22	0.219
FRE score	52.80 ± 10.14	52.71 ± 9.93	53.45 ± 9.80	0.713
QAMAI score	18.22 ± 0.85	18.17 ± 1.19	18.11 ± 1.35	0.418
Quora—“Periodontology”				
FKGL score	11.86 ± 1.45	11.07 ± 1.88	10.19 ± 1.92	0.096
FRE score	41.96 ± 8.99	32.14 ± 9.25	38.36 ± 10.17	0.177
QAMAI score	18.46 ± 1.35	18.23 ± 1.51	19.13 ± 1.39	0.127

^∗^ *p* value <0.05 subgroup analyses were exploratory, and *p* values were not adjusted for multiple comparisons.

Across domains, the most “readable” cluster (lowest FKGL) was observed for “gum recession,” especially for Gemini (FKGL 8.22 ± 1.22). Conversely, “periodontal regeneration” consistently produced higher grade‐level text across all models (FKGL 11.10–11.73), suggesting that this topic elicited more technical phrasing even when the question source was patient‐generated.

For FRE, the “gum recession” subgroup showed the highest readability with scores always above 50, 52.80 for ChatGPT‐4o, 52.71 for DeepSeek‐R1, and 53.45 for Gemini. The Quora “Periodontology” group had the lowest readability scores ranging between 32.14 (DeepSeek) and 41.96 (ChatGPT).

QAMAI values across keyword domains ranged from 17.14 to 20.38. The lowest mean QAMAI scores were observed in the ”pockets” subgroup (ChatGPT‐4o 17.59 ± 2.54, DeepSeek‐R1 17.14 ± 2.21, Gand emini 18.09 ± 2.63), placing two models (ChatGPT‐4o and DeepSeek‐R1) at the upper end of the “Fair quality” band (12–17) and Gemini within the ”Good quality” band (18–23). The mean scores of all other responses always fell within the “good quality” range, with the highest QAMAI means recorded for ”periodontitis” (ChatGPT‐4o 20.20 ± 1.33, Gemini 20.11 ± 1.47) and for ”periodontal regeneration” with Gemini (20.38 ± 1.29).

## 4. Discussion

The present study aimed to evaluate the performance of three LLMs, ChatGPT‐4o, Google Gemini 2.0 flash, and DeepSeek‐R1, in responding to patient‐centered inquiries regarding periodontology. While no statistically significant differences were found among the LLMs regarding the clinical quality and accuracy (QAMAI) of the responses (*p* = 0.078), significant differences were observed in terms of readability, specifically in the FKGL scores (*p* = 0.007) (Table 1).

Beyond mere statistics, however, it is necessary to consider the results obtained by LLMs in absolute terms, which provide an indication of the quality and readability of their output, the primary objective of this study. The qualitative assessment using the QAMAI tool yielded mean scores ranging from 18.31 (Google Gemini) to 19.10 (DeepSeek‐R1). According to the validation study by Vaira et al. [26], scores falling within the 18–23 range are classified as “Good Quality,” indicating that the information is mostly reliable and complete, though with some areas for refinement. It is noteworthy that none of the models achieved “Very Good” or “Excellent Quality” scores (scores >24), suggesting that although these tools may provide useful general information, they currently lack the nuance and comprehensiveness of a specialist consultation. This finding aligns with the systematic review by Alhazmi et al. [1], which concluded that while LLMs hold promise as supplementary tools, concerns about occasional inaccuracies necessitate verification against validated sources.

Interestingly, the newly introduced DeepSeek‐R1 achieved the highest absolute mean QAMAI score, although the difference was not statistically significant compared to that of the established competitors. This mirrors recent findings in implantology by Tuzlali et al. [9], where DeepSeek‐R1 demonstrated strong potential, performing comparably to ChatGPT‐o1 in accuracy and relevance domains.

Othman et al. [4] found that GPT‐4 significantly outperformed GPT‐3.5 in accuracy across dental specialties, emphasizing that model architecture updates play a critical role in performance. The fact that our study utilized the free versions of these models to simulate a realistic patient scenario might explain why the scores did not reach the “Excellent” tier often seen with paid, advanced models in other studies [30].

A critical finding of this study is the low readability of the AI‐generated responses. The mean FKGL scores ranged from 9.84 (DeepSeek) to 11.18 (ChatGPT‐4o), indicating that the content requires a high‐school to college‐level education to be comprehended. This significantly exceeds the recommended 6th to 8th‐grade reading level for patient education materials [31, 32]. This issue is consistent across the dental disciplines. Guven et al. [6] reported similar findings in dental traumatology, where AI responses were classified as “difficult to read” with FKGL scores often exceeding the 11th‐grade level. Similarly, Prasad et al. [10] found that in maxillofacial prosthodontics, ChatGPT produced the highest FKGL scores, while DeepSeek generated slightly more readable content, a trend confirmed by our data, where DeepSeek exhibited the lowest FKGL. This complexity may therefore act as a barrier to understanding for most patients who turn to an LLM to ask questions about their periodontal health, and it could create additional confusion for the user rather than helping to clarify their concerns [11].

Regarding the FRE, the mean scores ranged from 42.37 (Google Gemini) to 49.55 (ChatGPT‐4o). According to the standard interpretation of the Flesch scale, scores between 30 and 49 are classified as “difficult,” implying that the text is best understood by individuals with a college education. These findings are consistent with the recent literature in other dental domains. Guven et al. [6] reported similar FRE scores (ranging from 46.42 to 51.91) in dental traumatology, classifying the AI responses as “difficult to read”. Despite the differences in their underlying architectures, all three models defaulted to a complex linguistic register, with no statistically significant difference in their mean FRE scores (*p* = 0.144). However, it is crucial to note the wide standard deviations (≈10–11 points) observed across all groups. This suggests noticeable variability in readability across individual answers even within the same model, indicating a lack of consistency in the complexity of the generated output. Consequently, while some individual responses may approach accessible levels, in aggregate, FRE scores confirm that current LLMs fail to reliably meet the recommended standard for patient education materials (scores of 60–70, “Standard Plain English”) without specific prompting to simplify language.

Our findings on the performance of LLMs in periodontology appear consistent with broader trends in dentistry, where LLMs consistently demonstrate the potential as supplementary educational tools. In endodontics, Dufey‐Portilla et al. [30] observed that while LLMs show high validity under lenient criteria, their reliability drops significantly under stricter clinical thresholds. In pediatric dentistry, Padmanabh et al. [33] reported that ChatGPT provided accurate and high‐quality responses regarding primary tooth eruption, with 75% of answers rated as adequate or acceptable by pediatric dentists. Similarly, in orthodontics, Alkhamees [34] observed that ChatGPT‐4 generated “good” quality information regarding complex topics like impacted canines and orthognathic surgery; however, the study emphasized that answers often lacked the precision required for individual case management and failed to provide references, reinforcing our conclusion that AI cannot replace professional opinion. The necessity for professional oversight is further supported by Aboalshamat et al. [7], who evaluated AI responses to oral health queries from pregnant women. While they found ChatGPT‐4o mini to be largely accurate and clear, specific hallucinations, such as the scientifically incorrect notion that fetal calcium is drawn from the mother’s teeth, underscored the critical need for clinician supervision to prevent the spread of misconceptions. Furthermore, regarding the comparison between models, our finding that Google Gemini is a strong competitor to ChatGPT is echoed by Yüceer‐Çetiner et al. [12] in the field of orthognathic surgery. They reported that Google Gemini actually outperformed GPT‐4 in terms of global quality, empathy, and clinical appropriateness, suggesting that the superior model may vary depending on whether the clinical scenario prioritizes technical readability or empathetic communication. In our study, dealing with unstructured patient queries, the performance gap between Google Gemini and ChatGPT was negligible. This suggests that while GPT‐4 may possess superior deductive reasoning for complex board‐style questions, its ability to generate helpful patient information is comparable among the leading models [16, 20].

From a clinical perspective, the most relevant implication is not that patients should be encouraged to rely independently on LLMs but that clinicians should recognize that many patients are already using them. Periodontal practices may therefore benefit from explicitly discussing AI‐generated information during consultations, recommending that patients ask for simplified explanations, and encouraging the verification of AI advice with dental professionals. Practical prompt strategies may include asking the model to “explain this at a sixth‐grade reading level,” to “avoid technical terms,” or to “list when I should see a dentist urgently.” Such strategies cannot guarantee accuracy, but they may improve accessibility and reduce misunderstandings.

The ethical implications of AI‐generated periodontal information also deserve emphasis [35, 36]. Even when the average quality score is acceptable, individual responses may omit clinically important warnings, provide overconfident reassurance, or fail to account for patient‐specific risk factors such as smoking, diabetes, tooth mobility, or previous periodontal breakdown. Because periodontitis requires diagnosis, staging, grading, and individualized treatment planning, AI‐generated answers should be framed as general educational support rather than clinical advice. Professional oversight remains essential to prevent misinformation from delaying diagnosis or care.

Despite the interesting results and implications that derive from them, this study has some limitations. First, the cross‐sectional design captures the performance of LLMs at a single point in time (February 2025); given the rapid evolution of these models—as noted by Umer et al. [13] regarding the constant updates from GPT‐3.5 to GPT‐4—results may quickly become outdated. Second, the dynamic nature of the search algorithms used by Reddit and Quora limits the complete reproducibility of the question‐retrieval process. Although predefined keywords, eligibility criteria, and independent screening were used, the total numbers of posts displayed, screened, and excluded were not prospectively recorded. The subgroup analyses should also be interpreted with caution because some keyword domains included small numbers of questions, particularly the “periodontal regeneration” subgroup. Furthermore, multiple comparisons were performed across outcomes and keyword domains without formal adjustment for multiplicity, increasing the risk of type‐I error. Subgroup‐specific findings should therefore be considered exploratory and hypothesis‐generating. Third, the exclusion of image‐based queries limits the assessment of multimodal capabilities, which are increasingly relevant in periodontology for interpreting radiographs or clinical photographs [5]. Fourth, the study was conducted in English; however, as Dündar Sari and Sezer [37] pointed out, LLM accuracy can vary significantly depending on the language used, with English typically yielding better results than other languages. Finally, the use of expert raters for QAMAI, although calibrated, retains an element of subjectivity compared to standardized testing [9]. This applies only to QAMAI, while for FKGL and FRE, the element of subjectivity is clearly absent.

## 5. Conclusions

Within the limitations of this study, the following conclusions can be drawn:

All tested LLMs tested achieved mean QAMAI scores at the lower end of the predefined good‐quality range, with no significant differences among models. They can serve as useful supplementary information sources but should not replace professional consultation.

A significant limitation of all tested AI models is the complexity of the language used, highlighting the need for prompt engineering strategies or model fine‐tuning to make dental information more accessible to the general public.

Future developments should focus on training new specific models to deliver more simplified, patient‐centric explanations and integrating multimodal capabilities to assist in visual self‐monitoring of periodontal health.

## Author Contributions

**Nicola Alberto Valente:** Conceptualization, methodology, supervision, investigation, writing – original draft, writing – review and editing, project administration. **Lorenzo Floris:** data curation, investigation, formal analysis. **Chiara Cinquini:** Writing – original draft and editing. **Zuhair S. Natto:** Formal analysis. **Lorenzo Mordini:** Investigation, validation, writing – review and editing.

## Funding

This study did not receive any specific funding. Open access publishing facilitated by Universita degli Studi di Cagliari, as part of the Wiley ‐ CRUI‐CARE agreement.

## Disclosure

All authors have read and approved the final version of the manuscript. Nicola Alberto Valente had full access to all of the data in this study and takes complete responsibility for the integrity of the data and the accuracy of the data analysis.

## Ethics Statement

Ethical approval was not required for this study as it did not involve human participants, clinical interventions, or identifiable personal data. All questions were retrieved from publicly accessible online platforms and were fully anonymized.

## Conflicts of Interest

The authors declare no conflicts of interest.

## Supporting Information

Additional supporting information can be found online in the Supporting Information section.

## Supporting information


**Supporting Information** Table S1: List of patient questions and corresponding responses generated by ChatGPT‐4o, Google Gemini 2.0 Flash, and DeepSeek‐R1.

## Data Availability

The authors confirm that the data supporting the findings of this study are available within the article and its Supporting Information.
